# A *bla*_SIM-1_ and *mcr-9.2* harboring *Klebsiella michiganensis* strain reported and genomic characteristics of *Klebsiella michiganensis*

**DOI:** 10.3389/fcimb.2022.973901

**Published:** 2022-08-24

**Authors:** Shuangshuang Li, Xiaoyuan Jiang, Cuidan Li, Yingjiao Ju, Liya Yue, Fangzhou Chen, Lingfei Hu, Jing Wang, Xin Hu, Bahetibieke Tuohetaerbaike, Hao Wen, Wenbao Zhang, Dongsheng Zhou, Zhe Yin, Fei Chen

**Affiliations:** ^1^ Chinese Academy of Sciences (CAS) Key Laboratory of Genome Sciences and Information, Beijing Institute of Genomics, Chinese Academy of Sciences and China National Center for Bioinformation, Beijing, China; ^2^ School of Future Technology, University of Chinese Academy of Sciences, Beijing, China; ^3^ College of Life Sciences, University of Chinese Academy of Sciences, Beijing, China; ^4^ State Key Laboratory of Pathogen and Biosecurity, Beijing Institute of Microbiology and Epidemiology, Beijing, China; ^5^ State Key Laboratory of Pathogenesis, Prevention and Treatment of High Incidence Diseases in Central Asia, Department of Respiratory Medicine, First Affiliated Hospital of Xinjiang Medical University, Urumqi, China; ^6^ Department of Respiratory Medicine, First Affiliated Hospital of Xinjiang Medical University, Urumqi, China; ^7^ Department of Respiratory Medicine, Second Affiliated Hospital of Hainan Medical University, Haikou, China

**Keywords:** *Klebsiella michiganensis*, drug resistance, carbapenemase, mobile colistin resistance (mcr), mobile elements, plasmid, *bla*_SIM-1_, antibiotics

## Abstract

As a newly emerging *Klebsiella* pathogen, more and more *Klebsiella michiganensis* drug resistant strains have been reported in recent years, which posed serious threats to public health. Here we first reported a multidrug-resistant *K. michiganensis* strain 12084 with two *bla*_SIM-1_ and one *mcr-9.2* genes isolated from the sputum specimen of a patient in the Second Affiliated Hospital of Zhejiang University School of Medicine and analyzed its genetic basis and drug-resistance phenotypes. Genetic analysis showed that this strain harbored three different incompatibility groups (IncHI2, IncHI5, and IncFII_pKPHS2_:IncFIB-4.1) of plasmids (p12084-HI2, p12084-HI5, and p12084-FII). A total of 26 drug-resistance genes belonging to 12 classes of antibiotics were identified, most of which (24) were located on two plasmids (p12084-HI2 and p12084-HI5). Interestingly, two *bla*_SIM-1_ genes were identified to locate on p12084-HI2 and p12084-HI5, respectively, both of which were embedded in In630, indicating their genetic homogeny. It was noting that one *bla*_SIM-1_ gene was situated in a novel unit transposon (referred to as Tn*6733*) on the p12084-HI5 plasmid. We also discovered an *mcr-9.2* gene on the p12084-HI2 plasmid. To the best of our knowledge, this is the first report of a *bla*_SIM-1_ and *mcr-9.2* harboring *K. michiganensis* strain. We then investigated the population structure/classification, and antibiotic resistance for all 275 availably global *K. michiganensis* genomes. Population structure revealed that *K. michiganensis* could be divided into two main clades (Clade 1 and Clade 2); the most popular ST29 was located in Clade 1, while other common STs (such as ST50, ST27, and ST43) were located in Clade 2. Drug-resistance analysis showed 25.5% of the *K. michiganensis* strains (70/275) harboring at least one carbapenemase gene, indicating severe drug resistance of *K. michiganensis* beyond our imagination; this is a dangerous trend and should be closely monitored, especially for ST27 *K. michiganensis* with the most drug-resistant genes among all the STs. Overall, we reported a *bla*_SIM-1_ and *mcr-9.2* harboring *K. michiganensis* strain, and further revealed the population structure/classification, and drug-resistance of *K. michiganensis*, which provided an important framework, reference, and improved understanding of *K. michiganensis*.

## Introduction

*Klebsiella* includes at least 27 species, such as *K. pneumoniae*, *K. quasipneumoniae*, *K. variicola*, *K. quasivariicola*, *K. africana*, *K. terrigena*, *K. huaxiensis*, *K. michiganensis*, and *K. oxytoca* (Merla et al., 2019; Wyres et al., 2020). *K. michiganensis* was originally discovered in a toothbrush holder in a Michigan household in 2012 (Saha et al., 2013). Until now, this potential emerging pathogen has been reported in clinical settings in many countries (Mediavilla et al., 2016; Chapman et al., 2020; Sands et al., 2021).

Importantly, in recent years, more and more drug resistant *K. michiganensis* strains, even carbapenem resistance ones, have been reported, which could lead to high morbidity and mortality (Xu et al., 2017), further causing great threats to global public health (De Oliveira et al., 2020). The prevalence of carbapenem resistance in *K. michiganensis* is mainly derived from carbapenemase genes on various types of drug resistance plasmids, such as *bla*_KPC_, *bla*_NDM_, *bla*_IMP_, and *bla*_VIM_ genes (Tzouvelekis et al., 2012). Some *K. michiganensis* strains have even been reported to carry more than one carbapenemase gene, such as co-harboring of *bla*_KPC-2_, *bla*_NDM-1,_ and *bla*_NDM-5_ (Zheng et al., 2018), and co-harboring of *bla*_IMP-4_ and *bla*_NDM-1_ (Li et al., 2021). The *bla*_SIM-1_ gene was first discovered in *Acinetobacter pittii* in a Korean hospital in 2003 (Lee et al., 2005), which could cause carbapenem resistance (Palzkill, 2013; Bean and Wareham, 2022), but it has not been reported in *K. michiganensis*.

Polymyxin is known to be the last line for fighting against carbapenem-resistant Gram-negative bacteria (Poirel et al., 2017). In November 2015, a plasmid-mediated polymyxin resistance gene *mcr*-1 was first discovered in *Escherichia coli* and *K. pneumoniae* from animal and human in China (Liu et al., 2016). In recent years, *mcr*-2 to *mcr*-10 have been reported worldwide (Wang et al., 2020). The global dissemination of *mcr* genes causes significant threats to the last-line of antibiotic defense. Notably, more and more multidrug-resistant (MDR) *klebsiella* species co-producing carbapenemases and MCR have been clinically isolated, resulting in severe clinical problems because of very limited options for the treatments of such cases (Kaye et al., 2016; Mendes et al., 2018; Wang et al., 2018; Campos-Madueno et al., 2021), but such MDR strain has not been reported in *K. michiganensis*.

In this study, we first reported an MDR *K. michiganensis* strain 12084 co-harboring carbapenemase gene *bla*_SIM-1_ and MCR gene *mcr*-*9.2*, and its genetic characteristics were comprehensively analyzed. Furthermore, we revealed the population structure/classification, and antibiotic resistance genes of *K. michiganensis* for the first time by analyzing all 275 available *K. michiganensis* genomes, which provided an important framework for *K. michiganensis*.

## Materials and methods

### Bacterial isolation and identification

Strain 12084 was isolated in 2013 from the sputum specimen of a patient from the Second Affiliated Hospital of Zhejiang University School of Medicine in Hangzhou City, China. Strain 12084 was initially misidentified as *K. oxytoca* by MALDI-TOF/MS and finally identified as *K. michiganensis* by average nucleotide identity (ANI) analysis using *Pyani-0.2.7* (Richter and Rossello-Mora, 2009).

### Antimicrobial susceptibility testing

*In vitro* antimicrobial susceptibility tests of ampicillin, ampicillin/sulbactam, piperacillin, piperacillin/tazobactam, cefazolin, cefuroxime, cefuroxime axetil, cefotetan, ceftazidime, ceftriaxone, aztreonam, imipenem, meropenem, amikacin, gentamicin, tobramycin, ciprofloxacin, levofloxacin, nitrofurantoin, and trimethoprim/sulfamethoxazole were performed using BioMérieux VITEK 2. The minimum inhibitory concentration (MIC) of polymyxin B was determined using the broth microdilution method following the recommendations of the Clinical and Laboratory Standards Institute (CLSI, 2020). All the above results of antimicrobial susceptibility test were interpreted according to the CLSI guidelines.

### Conjugal transfer

Conjugal transfer experiments were carried out with the rifampin-resistant *Escherichia coli* EC600 used as a recipient and the *bla*_SIM_-positive 12084 isolate as a donor. Three milliliters of overnight cultures of each donor and recipient bacteria were mixed together, harvested and resuspended in 80 μL of Brain Heart Infusion (BHI) broth (BD Biosciences). The mixture was spotted on a 1 cm^2^ hydrophilic nylon membrane filter with a 0.45 µm pore size (Millipore) that was placed on BHI agar (BD Biosciences) plate and then incubated for mating at 22 °C for 24 h. Bacteria were washed from filter membrane and spotted on Muller-Hinton (MH) agar (BD Biosciences) plates containing 2500 μg/mL rifampin together with 4 μg/mL meropenem for selecting an *E. coli* transconjugant carrying *bla*_SIM_ (p12084-HI2 and p12084-HI5).

### Sequencing and sequence assembly

Genomic DNA was isolated from 12084 isolate using a Qiagen blood & cell culture DNA maxi kit. Genome sequencing was performed with a sheared DNA library with an average size of 15 kb (ranging from 10 kb to 20 kb) on a PacBio RSII sequencer (Pacific Biosciences, CA, USA), as well as a paired-end library with an average insert size of 400 bp (ranged from 150 kb to 600 kb) on a HiSeq sequencer (Illumina, CA, USA). *De novo* assembly was performed using the Hierarchical Genome Assembly Process 3 (HGAP3) within SMRT Analysis v2.3.0 (https://smrt-analysis.readthedocs.io/en/latest/). Adapters and low-quality sequences were trimmed and filtered using *FASTX*-*Toolkit* (http://hannonlab.cshl.edu/fastx_toolkit/).

### Sequence annotation and bioinformatics

Open reading frames (ORFs) and pseudogenes were predicted using *RAST* 2.0 (Brettin et al., 2015) combined with *BLASTP/BLASTN* searches (Boratyn et al., 2013) against the *UniProtKB/Swiss-Prot* database (Boutet et al., 2016) and the *RefSeq* database (O’Leary et al., 2016). Annotations of resistance genes, mobile elements, and other features were carried out using the online databases including *CARD* (Jia et al., 2017), *ResFinder* (Zankari et al., 2012), *ISfinder* (Siguier et al., 2006), *INTEGRALL* (Moura et al., 2009), and *Tn Number Registry* (Roberts et al., 2008). Multiple and pairwise sequence comparisons were performed using *MUSCLE* 3.8.31 (Edgar, 2004) and *BLASTN*, respectively. Gene organization diagrams were drawn in *Inkscape* 0.48.1 (https://inkscape.org/en/). Multi locus sequence typing (MLST) analysis for *K. michiganensis* was performed through *mlst* (https://github.com/tseemann/mlst) based on the seven housekeeping genes (*gapA*, *infB*, *mdh*, *pgi*, *phoE*, *rpoB*, and *tonB*) from the PubMLST database (https://pubmlst.org/organisms/klebsiella-oxytoca/).

### Phylogenetic analysis

All the publicly available genomes of *K. michiganensis* isolates (n=306, updated on September 9, 2021) were downloaded. ANI analysis of the 306 isolates was performed against the 11 reference strains of *Klebsiella* spp. (*K. pneumoniae* HS11286, CP003200.1; *K. aerogenes* Ka37751, CP041925.1; *K. Africana* 200023, CP084874.1; *K. grimontii* 4928STDY7071328, LR607336.1; *K. huaxiensis* WCHKl090001, CP036175.1; *K. oxytoca* FDAARGOS_335, CP027426.1; *K. pasteurii* SB6415; *K. quasipneumoniae* KqPF26, CP065838.1; *K. quasivariicola* 08A119, CP084768.1; *K. variicola* FH-1, CP054254.1; *K. michiganensis* THO-011, NZ_AP022547.1). 275 K*. michiganensis* strains (274 publicly available strains and strain 12084) were mapped to the *K. michiganensis* reference strain THO-011, and single nucleotide polymorphisms (SNPs) were identified by *Mummer* v4 (Marçais et al., 2018). We filtered all the SNPs in the repetitive DNA regions as identified by *RepeatMasker* (http://www.repeatmasker.org/) and those in the mobile genetic elements including insertion sequences (ISs), transposons, integrons, and phage-related genes. Based on the final 503,605 SNPs, a maximum-likelihood clustering tree of the 275 global *K. michiganensis* isolates was constructed using *FastTree* V2.1.9 (Price et al., 2009) with a bootstrap value of 500 and visualized using iTOL v6 (https://itol.embl.de/).

### Nucleotide sequence accession numbers

The 12084-chromosome, p12084-HI2, p12084-HI5, and p12084-FII sequences were submitted to GenBank under accession numbers CP072119, MW148604, MW810613, and MZ398018, respectively.

## Results and discussion

### Case report

On July 11, 2013, a 44-year-old man, who had performed a kidney transplant seven years ago, was admitted to The Second Affiliated Hospital Zhejiang University School of Medicine, due to lung infectious lesion accompanied with diarrhea, fever, cough, and acratia. After being treated with multiple antibiotics in the hospital for 27 days, the patient was not recovered and volunteered for discharge. A *K. michiganensis* strain named 12084 and an *Acinetobacter baumannii* strain were isolated from the sputum specimens during routine sampling and culturing on the 16th day of hospitalization. Since the sputum bacterial cultures of this patient were performed since the beginning of hospitalization, we had reason to infer that the two strains were both acquired through nosocomial infection. Here, the antimicrobial susceptibility tests showed that the *A. baumannii* strain was susceptible to carbapenems, while the *K. michiganensis* strain 12084 was resistant to carbapenems and selected for further analysis. The strain 12084 was initially identified as *K. oxytoca* through MALDI-TOF/MS and further identified as an MDR strain, displaying resistance to 13 antibiotics belonging to six categories: penicillins (ampicillin, piperacillin, ampicillin/sulbactam, and piperacillin/tazobactam), cephalosporins I (cefazolin), cephalosporins II (cefuroxime axetil, cefotetan, and cefuroxime), cephalosporins III (ceftriaxone and ceftazidime), carbapenems (meropenem and imipenem), and sulfanilamides (trimethoprim/sulfamethoxazole) (**Table 1**). It also showed intermediate resistance to 3 antibiotics, including furanes (nitrofurantoin), fluoroquinolones (levofloxacin), and polymyxin B. This strain was susceptible to aminoglycosides (amikacin, gentamicin, and tobramycin), fluoroquinolones (ciprofloxacin), and monobactams (aztreonam) (**Table 1**).

**Table 1:** Antimicrobial drug susceptibility profiles.

Antibiotics	MIC (mg/L)
Ampicillin	>=32/R
Ampicillin/sulbactam	>=32/R
Piperacillin	>=128/R
Piperacillin/tazobactam	8/R
Cefazolin	>=64/R
Cefuroxime	>=64/R
Cefuroxime Axetil	>=64/R
Cefotetan	>=64/R
Ceftazidime	>=64/R
Ceftriaxone	>=64/R
Aztreonam	2/S
Imipenem	8/R
Meropenem	8/R
Amikacin	<=2/S
Gentamicin	4/S
Tobramycin	4/S
Ciprofloxacin	<=0.25/S
Levofloxacin	1/I
Nitrofurantoin	64/I
Trimethoprim/sulfamethoxazole	>=320/R
Polymyxin B	2/I

S, sensitive; R, resistant; I, intermediate.

### Species identification and genomic characteristics of the MDR strain

To analyze the genetic basis of strain 12084, whole-genome sequencing was performed using the long-read PacBio RSII sequencing platform (Pacific Biosciences, CA, USA) (**Table S1**) and short-read Illumina HiSeq sequencing platform (Illumina, CA, USA) (**Table S2**). The strain 12084 was further identified as *K. michiganensis* rather than *K. oxytoca* through ANI analysis among all available *Klebsiella* reference genomes. It showed the highest ANI value of 98.9% compared with *K. michiganensis* reference THO-011, while displayed lower ANI values (< 95%) with other reference genomes (**Table S3** **;** **Figure S1**).

**Figure 1:**
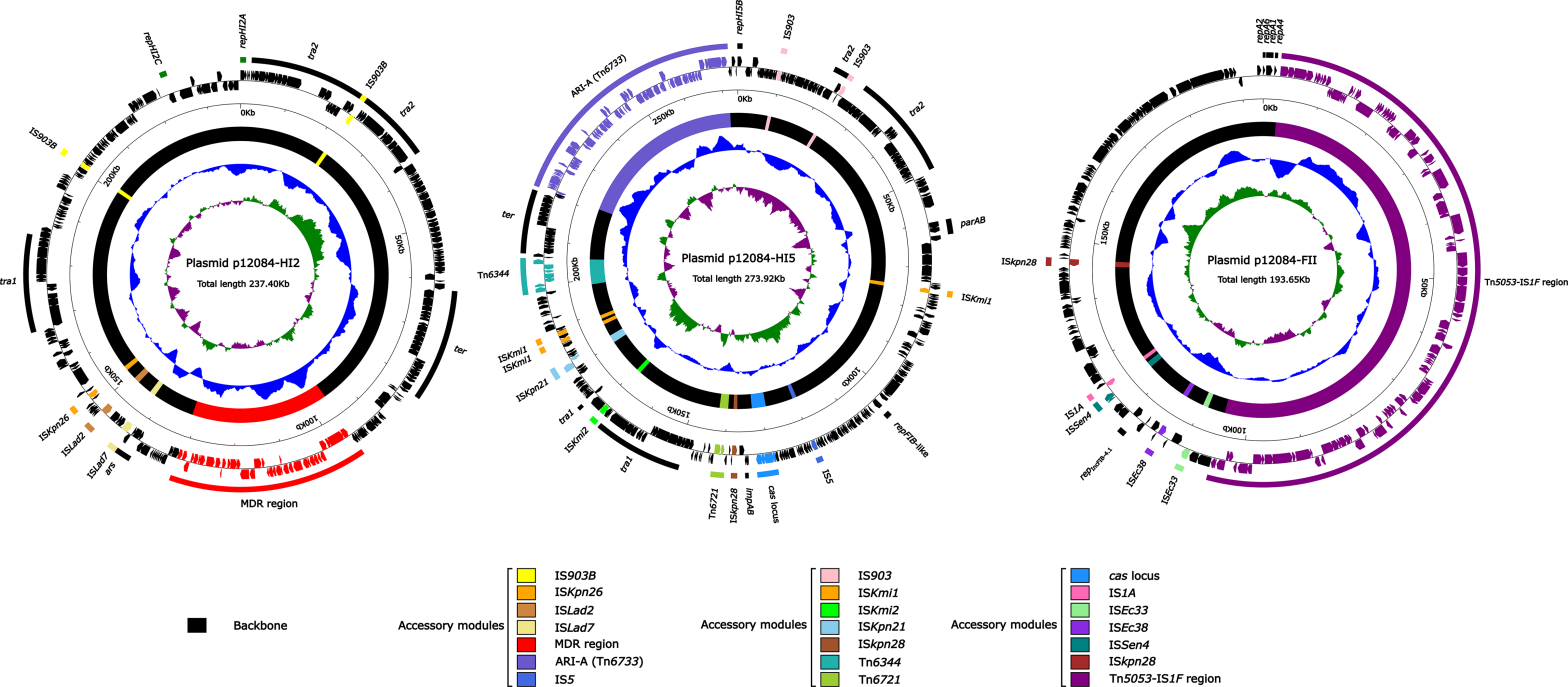
Schematic maps of p12084-HI2, p12084-HI5, and p12084-FII plasmids. Genes are denoted by arrows, and the backbone and accessory module regions are highlighted in black and color, respectively; The innermost circle presents GC-skew [(G-C)/(G+C)], with a window size of 500 bp and a step size of 20 bp. Inward indicates the relative content G > C, and outward indicates the relative content G < C; The next-to-innermost circle presents G and C content. Inward indicates below average G and C content, and outward indicates above average G and C content; The outermost circle shows the arrangement of genes, with genes forward and reverse in clockwise and counterclockwise directions, respectively.

Sequencing results showed that strain 12084 contained a ~6.2Mb circular chromosome and three plasmids. The three plasmids were designated as p12084-HI2, p12084-HI5, and p12084-FII, which had circularly closed DNA sequences of 237,396 bp, 273,923 bp, and 193,645 bp in length with mean G+C contents of 45.9%, 46.6%, and 51.7%, and predicted ORFs of 302 bp, 393 bp, and 268 bp, respectively (**Table 2**). These plasmids belonged to IncHI2, IncHI5, and IncFII_pKPHS2_:IncFIB-4.1 groups, respectively, because they contained the replication genes of *repHI2A* + *repHI2C*, *repHI5B* + *repFIB*-like, and *repA2*-*6-1*-*4*_IncFIIpKPHS2_ + *rep*_IncFIB-4.1_, respectively (**Figure 1**). Each plasmid was composed of backbone regions, together with accessory modules that were recognized with some acquired DNA regions with various mobile elements at different sites (**Table 2** **;** **Figure 1**).

**Table 2:** Major features of three plasmids from strain 12084.

Category	Plasmids		
	p12084-HI2	p12084-HI5	p12084-FII
Incomparability group	IncHI2	IncHI5	IncFII_pKPHS2_:IncFIB-4.1
Accession number	MW148604	MW810613	MZ398018
Total length (bp)	237,396	273,923	193,645
Total number of ORFs	302	393	268
Mean G+C content, %	45.9	46.6	51.7
Length of the backbone (bp)	195,609	197,719	85,363
Accessory modules	MDR region^#^, IS*Lad7*, IS*Lad2*, IS*Kpn26*, and IS*903B*	ARI-A (Tn*6733*) region ^#^, Tn*6721*, Tn*6344*, IS*Kpn21*, IS*5*, IS*kpn28*, IS*Kmi2*, IS*903*, and IS*Kmi1*	Tn*5053*-IS*1F* region, IS*Ec33*, IS*Ec38*, IS*Sen4*, IS*1A*, and IS*kpn28*

#, accessory modules containing resistance genes as listed in **Table 3**.

Both p12084-HI2 and p12084-HI5 owned large backbone regions and small accessory modules with a Tn*1696* structure. Here, the accessory modules of p12084-HI2 consisted of one MDR region and several ISs), and those of p12084-HI5 contained a new MDR region (designated as Tn*6733*), Tn*6721*, Tn*6344*, and several ISs (IS*903*/IS*kmi1*/IS*kmi2*/IS*kpn21*/IS*kpn28*) (**Figure 1**). P12084-FII had small backbone regions with a new large accessory module (named as Tn*5053*–IS*1F* region) (**Table 2** **;** **Figure 1**).

### Drug resistance genotype of *K. michiganensis* 12084

To illustrate the drug-resistance genotype of the *K. michiganensis* 12084, we identified drug resistance genes by *BLAST* against the database *ResFinder* (Zankari et al., 2012). A total of 26 drug resistance genes were identified, including aminoglycoside [*aph*(3’)-Ia, *aadA15e*, *aadA2*, and *armA*], β-lactam (*bla*_CTX-M-9_ and *bla*_OXY-1_), carbapenem (*bla*_SIM-1_), colistin (*mcr*-9.2), disinfectant (*qacED1*), fosfomycin (*fosA3*), macrolide [*mph*(E) and *msr*(E)], phenicol (*catB3*), quinolone (*qnrA1*), rifampicin (*ARR*-3), sulphonamide (*sul1*), and trimethoprim (*dfrA16*) (**Table 3**). Importantly, most drug resistance genes (92%; 24/26) were located on the accessory modules of the two MDR plasmids (p12084-HI2 and p12084-HI5) (**Table 3**). Here, the resistant phenotypes for all the above mentioned antibiotics of 12084 strains could be explained by the existence of corresponding drug resistant genes. In addition, although we identified some drug resistant genes related to aminoglycosides and fluoroquinolones (*aph*(3’)-Ia, *aadA15e*, *aadA2*, and *armA* for aminoglycosides; *qnrA1* for fluoroquinolones), the strain 12084 was susceptible to these two drugs. Previous studies have revealed that the two drug resistant genes on the plasmids produce very low levels of resistance for aminoglycosides and fluoroquinolones. (Rodríguez-Martínez et al., 2011; Saravolatz and Stein, 2020).

**Table 3:** Resistance genes in strain 12084.

Location	Resistance marker	Resistance phenotype	Position	Region
12084-chromosome	*aph*(3’)-Ia	Aminoglycoside resistance	3202195.3203008	
	*bla*_OXY-1_	β-lactam resistance	4745313.4746188	
p12084-HI2	*bla*_SIM-1_	Carbapenem resistance	102257.102997	MDR region
	*ARR-3*	Rifampicin resistance	103149.103601	
	*catB3*	Phenicol resistance	103750.104382	
	*aadA15e*	Aminoglycoside resistance	104440.105231	
	*qacED1*	Disinfectant resistance	105395.105742	
	*sul1*	Sulphonamide resistance	105736.106575	
	*qnrA1*	Quinolone resistance	108785.109441	
	*sul1*	Sulphonamide resistance	111093.111932	
	*mcr-9.2*	Colistin resistance	117145.118767	
	*bla*_CTX-M-9_	β-lactam resistance	122764.123639	
	*sul1*	Sulphonamide resistance	125912.126778	
	*qacED1*	Disinfectant resistance	126745.127092	
	*aadA2*	Aminoglycoside resistance	127256.128035	
	*dfrA16*	Trimethoprim resistance	128152.128625	
p12084-HI5	*fosA3*	Fosfomycin resistance	227235.227651	ARI-A (Tn*6733*) region
	*mph*(E)	Macrolide resistance	251162.252046	
	*msr*(E)	Macrolide resistance	252102.253577	
	*armA*	Aminoglycoside resistance	255876.256623	
	*sul1*	Sulphonamide resistance	261084.261923	
	*qacED1*	Disinfectant resistance	261917.262264	
	*aadA15e*	Aminoglycoside resistance	262428.263219	
	*catB3*	Phenicol resistance	263277.263909	
	*ARR-3*	Rifampicin resistance	264058.264510	
	*bla*_SIM-1_	Carbapenem resistance	264662.265402	

It was noted that two copies of *bla*_SIM-1_ genes were observed on two plasmids (p12084-HI2 and p12084-HI5) of the strain, respectively. A colistin resistant gene *mcr-9.2* was also identified on p12084-HI2 (**Table 3**). To our best knowledge, this is the first reported *K. michiganensis* strain with *bla*_SIM-1_ and *mcr-9.2* genes.

### Genetic characteristics of the backbone regions of the two MDR plasmids from *K. michiganensis* 12084

Since most antibiotic resistance genes (ARGs) were located on the two MDR plasmids (p12084-HI2 and p12084-HI5), we further conducted a genetic characteristic analysis of both plasmids. The backbones of p12084-HI2 and p12084-HI5 were 195.6 kb and 197.7 kb in length, accounting for 82.4% and 72.2% of the plasmid genomes, respectively.

The p12084-HI2 backbone showed 79% *BLAST* query coverage and 99% nucleotide identity to the IncHI2 reference plasmid R478 (GenBank accession no. BX664015) (Gilmour et al., 2004). These two plasmids shared the core IncHI2 backbone markers, including *repHI2A* and *repHI2C* for replication initiation, the *tra1* and *tra2* regions for conjugal transfer, and the *ter* region for tellurium resistance, and the *ars* locus for arsenic resistance (**Figure S2A**). On the other hand, three major differences were identified between the two backbone sequences: IS*903B* was inserted between *parB2* and *htdA* within the *tra2* region of p12084-HI2; compared to R478, MDR region of p12084-HI2 was inserted at a site downstream of *dcm*, leading to the truncation of *dcm* and deletion of *orf819* to *orf168* region; another IS*903B* was inserted at a site downstream of *orf450*, resulting in the truncation of *orf450* and loss of *orf198* to *orf2385* region (**Figure S2A**).

The p12084-HI5 backbone had 86% *BLAST* query coverage and 99% nucleotide identity to the IncHI5 reference plasmid pA324-IMP (GenBank accession no. CP026017) (Liang et al., 2019), with conserved IncHI5 backbone genes or gene loci (including *repHI5B* together with its iterons and *repFIB*-like for replication, *parABC* for partition, *tra1* and *tra2* for conjugal transfer, and *ter* for tellurium resistance) (**Figure S2B**). On the other hand, four different important events associated with gene acquisition/loss were identified between the backbone regions of these two plasmids. Firstly, IS*903* was inserted within *tivF6* (F-type type IV secretion, mating pair stabilization protein) across the *tra2* region in p12084-HI5; secondly, IS*Kmi2* was inserted within *orf3351* (superfamily I DNA helicase) across the *tra1* region in p12084-HI5; Thirdly, compared to pA324-IMP (reference), p12084-HI5 had underwent the truncations of *impB* and *umuC2* genes, and the deletion of the backbone genes of the *thiF1* to *umuC1* region. Importantly, two different ARI-As (Tn*6733* from p12084-HI5, and Tn*6382* from pA324-IMP) carried different drug-resistance profiles, although they were both inserted at a site between *relB* and *orf342* (**Figure S2B**).

### The MDR regions in p12084-HI2 and p12084-HI5

**Figure 2:**
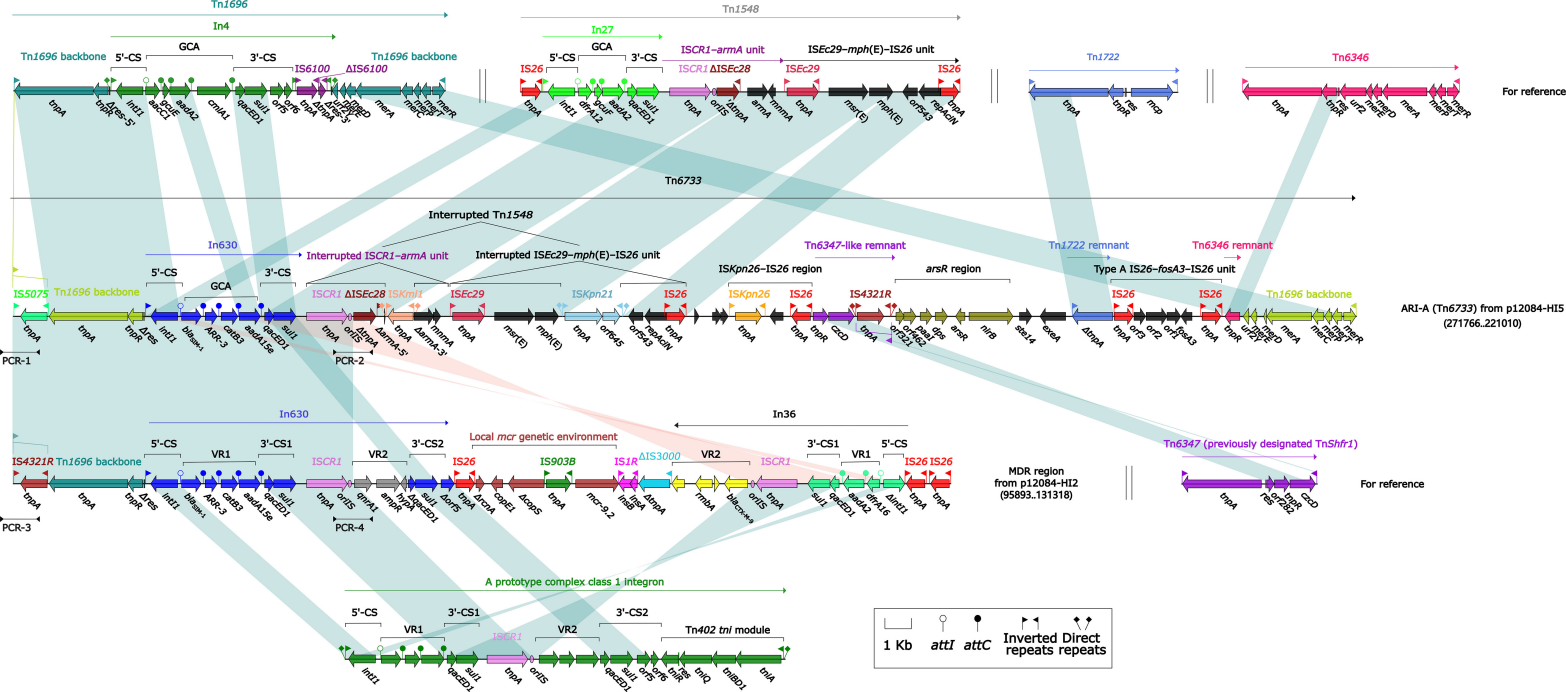
Organizations of the MDR regions of p12084-HI2, and p12084-HI5 compared with related reference regions. Genes are denoted by arrows. Genes, mobile elements, and other features are colored based on function classification. Shading denotes regions of homology (>95% nucleotide identity). Numbers in brackets indicate the nucleotide positions within the corresponding plasmids. The arrowheads indicate the locations of PCR primers and the expected amplicons. The accession numbers of Tn*1696*, Tn*1548*, Tn*1722*, Tn*6346*, and Tn*6347* for reference are U12338, AF550415, X61367, EU696790, and CP000447, respectively.

We further focused on the MDR regions to explore the genetic environment of antibiotic-resistant genes for the two plasmids. The MDR region (41.7 kb) from p12084-HI2 and the Tn*6733* (ARI-A island, 76.2 kb) from p12084-HI5 (**Figure 2**) were different derivatives of Tn*1696*. Tn*1696*, a unit transposon belonging to the Tn*21* subgroup of Tn*3*, was generated from the insertion of a class 1 integron In4 into the resolution (*res*) site of a primary backbone structure: IRL (inverted repeat left)–*tnpA* (transposase)–*tnpR* (resolvase)–*res*–*mer* (mercury resistance locus)–IRR (inverted repeat right) (Partridge et al., 2001).

The MDR region from p12084-HI2 (**Figure 2**) contained a Tn*1696*-resembling backbone region with IRL-*tnpA*-*tnpR-*Δ*res* at the 5’-end, and its IRL was interrupted by IS*4321R* (Partridge and Hall, 2003). But it was not an intact transposon due to its absence of IRR at the 3’-end, which was derived from the insertion of the integron-associated regions [namely In630, the *mcr* genetic environment (carrying *mcr-9.2*), In36, and five independent ISs (including IS*1R*, ΔIS*3000*, and three copies of IS*26*)]. Both In630 and In36 from the MDR region belonged to complex class 1 integrons (Toleman et al., 2006), which harbored two intrinsic resistance regions, namely the cassette array *bla*_SIM-1_–*ARR-3*–*catB3*–*aadA15e* and *qnrA1* (In630), and the cassette array *dfrA16*–*aadA2* and *bla*_CTX-M-9_ (In36).

Similar to Tn*1696*, the ARI-A island (Liang et al., 2019) from p12084-HI5 had paired terminal 38-bp IRL/IRR, and thus it was identified as a novel unit transposon and designated as Tn*6733* (the highest coverage of 82% and identity of 99% compared to all available DNA sequences in the GenBank). ARI-A (Tn*6733*) differed from Tn*1696* in two aspects: i) In4 of Tn*1696* was replaced with some insertions at the same position within *res*, including a concise class 1 integron In630 (Sajjad et al., 2011) (the cassette array *bla*_SIM-1_–*ARR-3*–*catB3*–*aadA15e*), an *mph*(E) and *msr*(E)-carrying interrupted Tn*1548*, an IS*Kpn26*-IS*26* region, a Tn*6347*-like remnant, an *arsR* region (arsenic resistance), a Tn*1722* remnant, a fosfomycin resistance unit type A IS*26*–*fosA3*–IS*26*, and a Tn*6346* remnant; ii) IRL was interrupted by IS*5075* that was a hunter of terminal IRs of Tn*21* subgroup transposons (**Figure 2**) (Partridge et al., 2001).

Interestingly, a highly similar repetition region (12.8 kb in length, 100% *BLAST* query coverage, and 99% nucleotide identity) was observed in both p12084-HI2 and p12084-HI5, which was validated by a set of PCR amplifications targeting four key jointing fragments of the repetition region (**Figure 2**; **Table S4**). The repetition region was comprised of a Tn*1696* backbone structure, with an IRL–IS*5075*/IS*4321R*–*tnpA*–*tnpR*–Δ*res*, an In630 (the cassette array *bla*_SIM-1_–*ARR-3*–*catB3*–*aadA15e*) and an IS*CR1*, which might be resulted from the homologous recombination between the two plasmids, further contributing to extensive dissemination of drug resistance genes among various MDR plasmids.

### The *mcr* genetic environment in p12084-HI2

**Figure 3:**
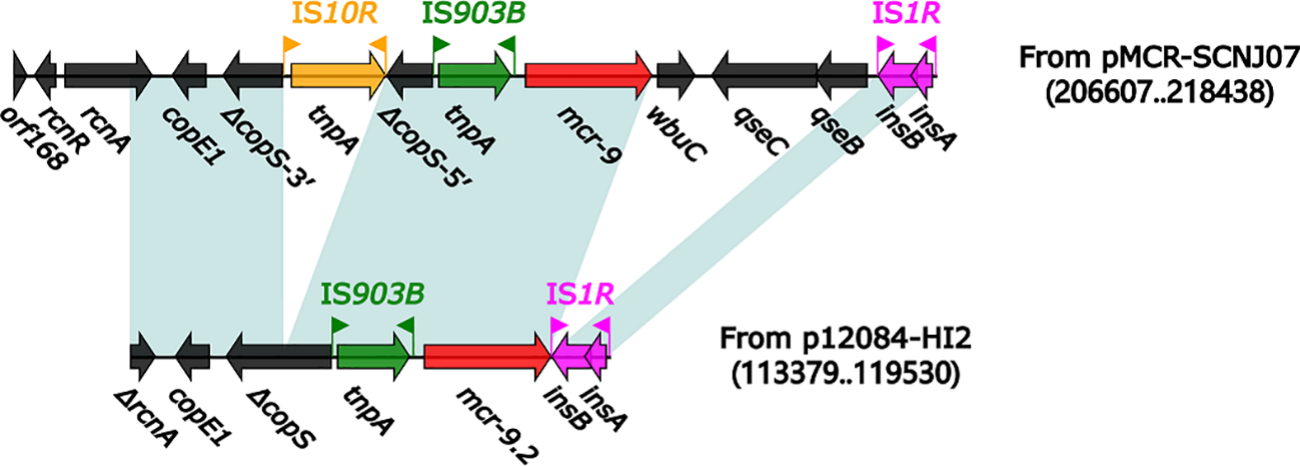
Organization of the *mcr* genetic environment from p12084-HI2 compared with related reference regions. Genes are denoted by arrows. Genes, mobile elements, and other features are colored based on function classification. Shading denotes regions of homology (>95% nucleotide identity). Numbers in parentheses indicate the nucleotide positions within the corresponding plasmids. The accession number of the pMCR-SCNJ07 is MK933279.

We then explored the genetic environment of *mcr-9.2* in p12084-HI2. The modular structure of the *mcr* genetic environment (6.2 kb in length) from p12084-HI2 (**Figure 3**) was organized sequentially as Δ*rcnA–copE1–*Δ*copS–*IS*903B–mcr*-*9.2–*IS*1R*, which showed partial homology with the *mcr* genetic environment in pMCR-SCNJ07 from the strain SCNJ07 (*rcnA–copE1–*Δ*copS*-3’*–*IS*10R–*Δ*copS*-5’*–*IS*903B–mcr-9–wbuC–qseC–qseB–*IS*1R*) (Yuan et al., 2019).

Compared to pMCR-SCNJ07, the *mcr* genetic environment of p12084-HI2 underwent the deletions of *wbuC–qseC–qseB* downstream of *mcr*-*9.2*, and IS*10R* within *copS*, and the truncation of *rcnA* (**Figure 3**). The difference in *mcr* genetic environment between the two plasmids might result in differential *mcr* drug resistances between the two strains: strain 12084 showed intermediate resistance to polymyxin B (MIC=2mg/L), while the strain SCNJ07 displayed resistance to polymyxin B (MIC=8 µg/mL). The modular structures of the *mcr-9* gene from ME-1 (Chavda et al., 2019), the *mcr-9* gene from LM22-1 (Khalifa et al., 2020), and the *mcr-9* gene from A2483 (Kananizadeh et al., 2020) all lack the *qseB* and *qseC* regulatory genes, which leads to the corresponding strains susceptible to colistin. Therefore, we speculated that strain 12084 showed intermediate resistance to polymyxin B due to the absence of *qseB* and *qseC* downstream of *mcr-9.2*, further indicating the importance of *the qseB/qseC* two-component system in *mcr-9* expression.

### Transferability

Both p12084-HI2 and p12084-HI5 could be transferred from the strain 12084 into *E. coli* EC600 through conjugation, generating the transconjugants 12084-SIM-EC600 (**Figure S3**). These were consistent with the presence of two complete sets of *tra1* and *tra2* genes in p12084-HI2 and p12084-HI5, making them self-transferable.

### Population structure/classification and antibiotic resistance of *K. michiganensis*

To further investigate the population structure/classification, and antibiotic resistance of *K. michiganensis*, a total of 306 K*. michiganensis* genomes were downloaded from NCBI (**Table S5**). ANI analysis showed that 274 (89.5%) strains belonged to *K. michiganensis* based on the *K. michiganensis* reference strain THO-011 (ANI value ≥ 95%), including 196 human-, 16 animal-, and one environment-isolates (**Table S5**). The remaining 32 strains were excluded from our study due to wrong classifications, among which 19 strains belonged to *K. grimontii* (ANI values were more than 99% compared with the *K. grimontii* reference strain 4928STDY7071328), 12 strains belonged to *K. pasteurii* (ANI values were more than 99% compared with the *K. pasteurii* reference strain SB6415), and one strain (RC10) did not belong to *Klebsiella* (ANI values were less than 95% compared with all the *Klebsiella* reference strains) (**Table S3** **;** **Figure S1**). These results indicated more precision classification for *K. michiganensis* through whole genome sequences.

**Figure 4:**
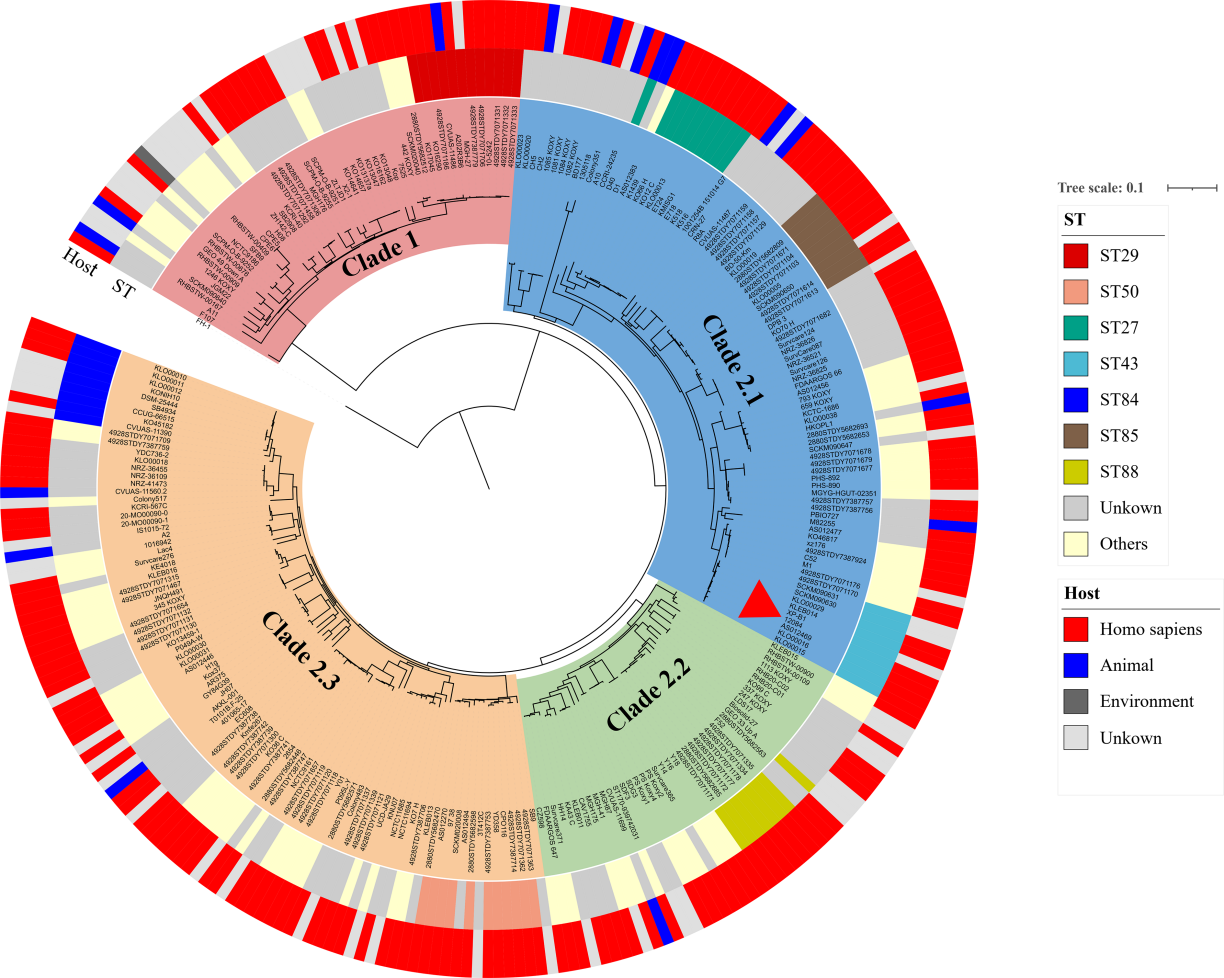
A maximum-likelihood clustering tree of the 275 available *K. michiganensis* strains. The maximum-likelihood clustering tree is constructed by 503,605 SNPs of all 275 K*. michiganensis* genome sequences. *K. variicola* FH-1 (GenBank accession no. CP054254) is used as the outgroup; The outermost circle presents the sources of the isolates; the sub-outside circle presents the STs of the isolates; the innermost circle presents different Clades of *K. michiganensis*; the strain 12084 is marked with a red triangle.

The phylogenetic tree was then constructed using the abovementioned 274 K*. michiganensis* strains and our MDR strain 12084. To the best of our knowledge, this is the first time to reveal the population structure of *K. michiganensis*. Here, *K. michiganensis* could be divided into two major clades on the tree: Clade1 and Clade2, which were also validated by ANI results (**Figure 4** **;** **Figure S1**). The Clade2 was further classified into three subclades: Clade2.1, Clade2.2, and Clade2.3. In addition, we performed MLST analysis on these *K. michiganensis* strains. The results showed that ST29 was the most common ST (12/275, 4.4%), followed by ST50 (11/275, 4%), ST27 (10/275, 3.6%), and ST43/ST84/ST85 (9/275, 3.3%) (**Figure 4**). The ST29 of *K. michiganensis* strains were located in Clade 1, while other STs (such as ST50, ST27, and ST43) were located in Clade 2. Notably, 120 isolates (43.6%) could not be assigned to known ST types. These results indicated genetic diversity and non-clonal transmission/population structure of *K. michiganensis* strains.

**Figure 5:**
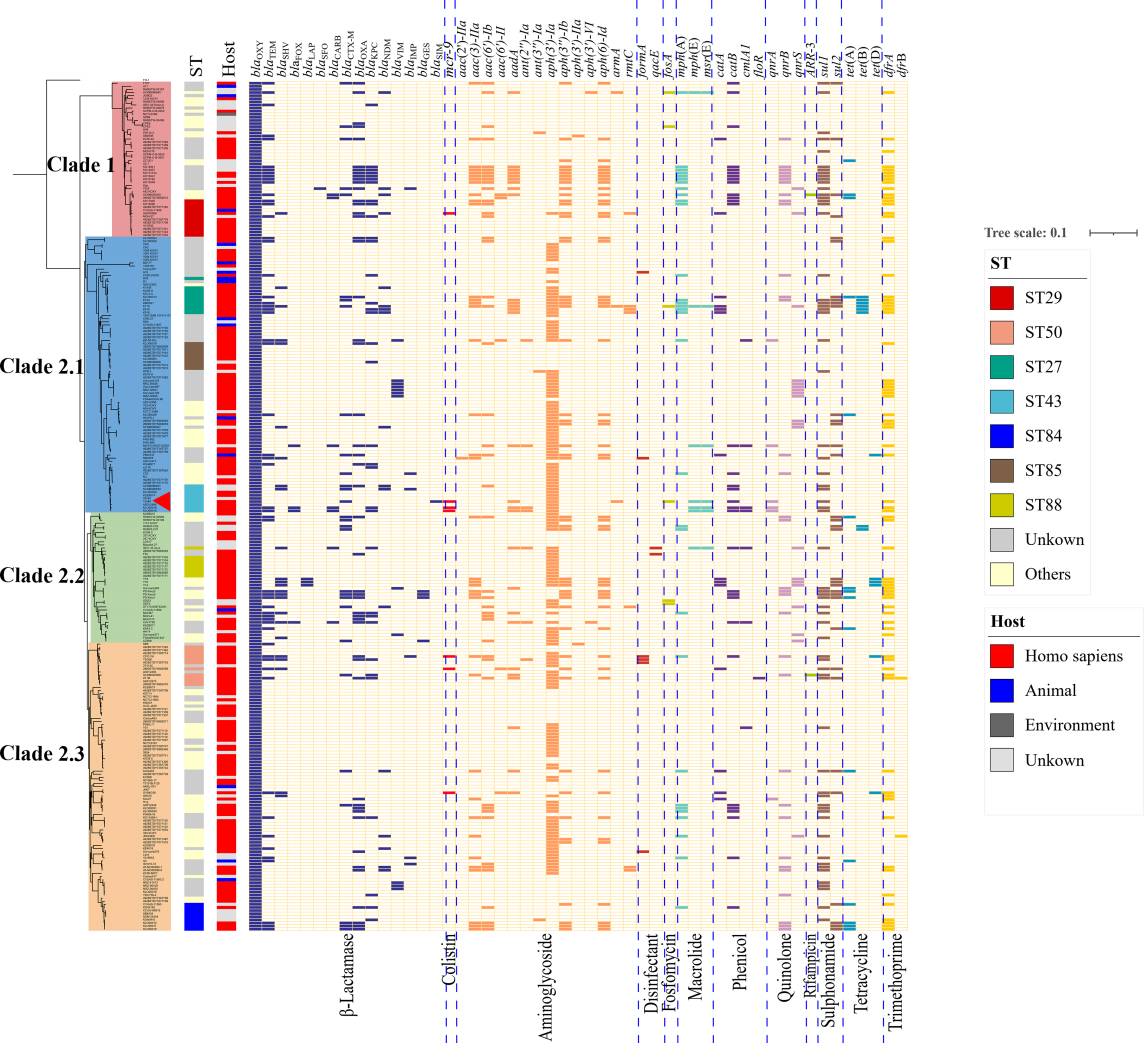
The maximum-likelihood tree and ARGs distributions in *K. michiganensis*. The matrix of ARGs is present for all *K. michiganensis* genomes against the dendrogram. The top of the matrix indicates the names of ARGs, while the bottom of that represents the groups of ARGs. The colored cells represent the “presence” of the ARGs, and the cells are colored based on the groups of ARGs. The strain 12084 is marked with a red triangle.

We also investigated the drug resistance profile of *K. michiganensis* strains. A total of 51 types of drug resistant genes were identified among these strains (**Figure 5**). Here, *bla*_OXY_ commonly existed on the chromosome of all *K. michiganensis* strains (275/275), ranking first, followed by *aph(3’)-Ia* (152/275, top-2) and *dfrA* (74/275, top-3) (**Figure 5**). We further determined the relationship between the drug-resistant gene number and Clades/STs. Our results revealed no significant difference for the drug-resistant gene number in different clades. However, the drug-resistant gene number showed a difference among different STs: ST27 isolates possess the most drug-resistant gene number among all the STs (**Figure S4A**), suggesting the potential threat of ST27 *K. michiganensis* strains to public health. This phenomenon should be closely monitored. Incidentally, we also compared the number of ARGs between human and animal. The results showed that human-derived isolates have a greater number of ARGs than animal-derived ones (*P* < 0.05; Wilcoxon rank test) (**Figure S4B**), which was consistent with previous studies (Brisse and Duijkeren, 2005).

Importantly, we further focused on carbapenem-resistant genes, since carbapenem resistance was a big problem in the treatment of *Klebsiella* strains (Hayden and Won, 2018). Our research showed that 25.5% of *K. michiganensis* strains (70/275) carried at least one carbapenemase gene, indicating severe carbapenem-resistance in *K. michiganensis* strains throughout the world; this is a dangerous tendency and should be closely monitored. Here, the 70 carbapenem-resistant *K. michiganensis* strains contained 34 *bla*_KPC_ genes, 17 *bla*_NDM_ genes, 12 *bla*_VIM_ genes, 6 *bla*_IMP_ genes, 4 *bla*_GES_ genes, and one *bla*_SIM_ gene (**Figure 5**). Further analysis demonstrated that *bla*_KPC_ preferred to be carried by ST27 (3, top-1), ST29 (3, top-1), and ST50 (3, top-1) strains, and *bla*_NDM_ preferred to be harbored by ST27 (4, top-1) strains (**Table S6**). These results also indicated the potential threat of ST27 *K. michiganensis* strains, and therefore constant surveillance for the dissemination of ST27 strains is warranted. In addition, it was worth noting that only one *bla*_SIM_ was identified in our strain 12084, indicating the emergence of the *bla*_SIM_-carrying *K. michiganensis* strain, which should be closely monitored.

## Conclusions

In summary, our findings provide important references and improved understanding of *K. michiganensis*. We first described the complete genome of a clinical MDR *K. michiganensis* strain 12084 with two *bla*_SIM-1_ and one *mcr-9.2* genes obtained from a Kidney transplant patient in China without a travel history. To the best of our knowledge, this is the first report of a *K. michiganensis* isolate with *bla*_SIM-1_ and *mcr-9.2* genes. We further revealed the antibiotic resistance genes, population structure/classification, and antibiotic resistance of *K. michiganensis* for the first time, and provided an important framework for *K. michiganensis*. The emergence of resistant *K. michiganensis* strains, especially for those MDR strains harboring carbapenemase genes or/and *mcr*, poses serious threats to public health, which is a dangerous trend and should be closely monitored.

## Data availability statement

The datasets presented in this study can be found in online repositories. The names of the repository/repositories and accession number(s) can be found in the article/**Supplementary Material**.

## Author contributions

FC, ZY, and DZ conceived the study and designed experimental procedures. XJ, WZ, FZC, and LY performed the experiments. SL, XJ, CL, YJ, and LH analyzed the data. JW, XH, BT, and HW contributed reagents and materials. FC, SL, XJ, and CL wrote the manuscript. All authors contributed to the article and approved the submitted version.

## Funding

This work was supported by Beijing Natural Science Foundation (Grant No. M21009), National Natural Science Foundation of China (NSFC) (Grant No. 31770870), Funds for International Cooperation and Exchange of the National Natural Science Foundation of China (Grant No. 32061143024).

## Conflict of interest

The authors declare that the research was conducted in the absence of any commercial or financial relationships that could be construed as a potential conflict of interest.

## Publisher’s note

All claims expressed in this article are solely those of the authors and do not necessarily represent those of their affiliated organizations, or those of the publisher, the editors and the reviewers. Any product that may be evaluated in this article, or claim that may be made by its manufacturer, is not guaranteed or endorsed by the publisher.

## Supplementary material

Supplementary Figure 1 Pairwise average nucleotide identity (ANI) cluster map of all the downloaded genomes with *Klebsiella* reference strains. Shown is the hierarchical clustering and heat map of pairwise ANI values among all strains. Their final species determination (*K. michiganensis*, *K. grimontii*, and *K. pasteurii*) are shown as colored bars adjacent to the heat map. The strain12084 is marked with a red triangle.

Supplementary Figure 2 Linear comparison of p12084-HI2 and p12084-HI5 with reference plasmids **(A)** Linear comparison of p12084-HI2 with the IncHI2 reference plasmid R478. Genes are denoted by arrows. Genes, mobile elements, and other features are colored based on function classification. Shading denotes regions of homology (>95% nucleotide identity). **(B)** Linear comparison of p12084-HI5 with the IncHI5 reference pA324-IMP. Genes are denoted by arrows. Genes, mobile elements, and other features are colored based on function classification. Shading denotes regions of homology (>95% nucleotide identity).

Supplementary Figure 3 PCR amplifications of *bla*_SIM,_ *repHI2A*, and *repHI5B* for 12084-SIM-EC600. 12084-SIM-EC600: the transconjugants. 12084 (the donor) and EC600 (the recipient) were used as the positive and negative control, respectively. *repHI2A* and *repHI5B* were the replication gene of p12084-HI2 and p12084-HI5, respectively. The 16S rDNA gene was used to identify the bacterial species of 12084-SIM-EC600.

Supplementary Figure 4 The ARG numbers in different STs/host isolates. **(A)**. Boxplot showed the top five ARG numbers in different STs isolates. The numbers on the y-axis represent the number of ARGs. **(B)**. Boxplot showed the numbers of ARGs in different host isolates. The numbers on the y-axis represent the number of ARGs. The *P* values were obtained using the Wilcoxon rank test. **, statistically significant with *P* < 0.01.
