# Supplementary figures and images for: A bla SIM-1 and mcr-9.2 harboring Klebsiella michiganensis strain reported and genomic characteristics of Klebsiella michiganensis
Source: Front Cell Infect Microbiol. 2022 Aug 24;12:973901. doi: 10.3389/fcimb.2022.973901 (PMC9448873; doi:10.3389/fcimb.2022.973901)

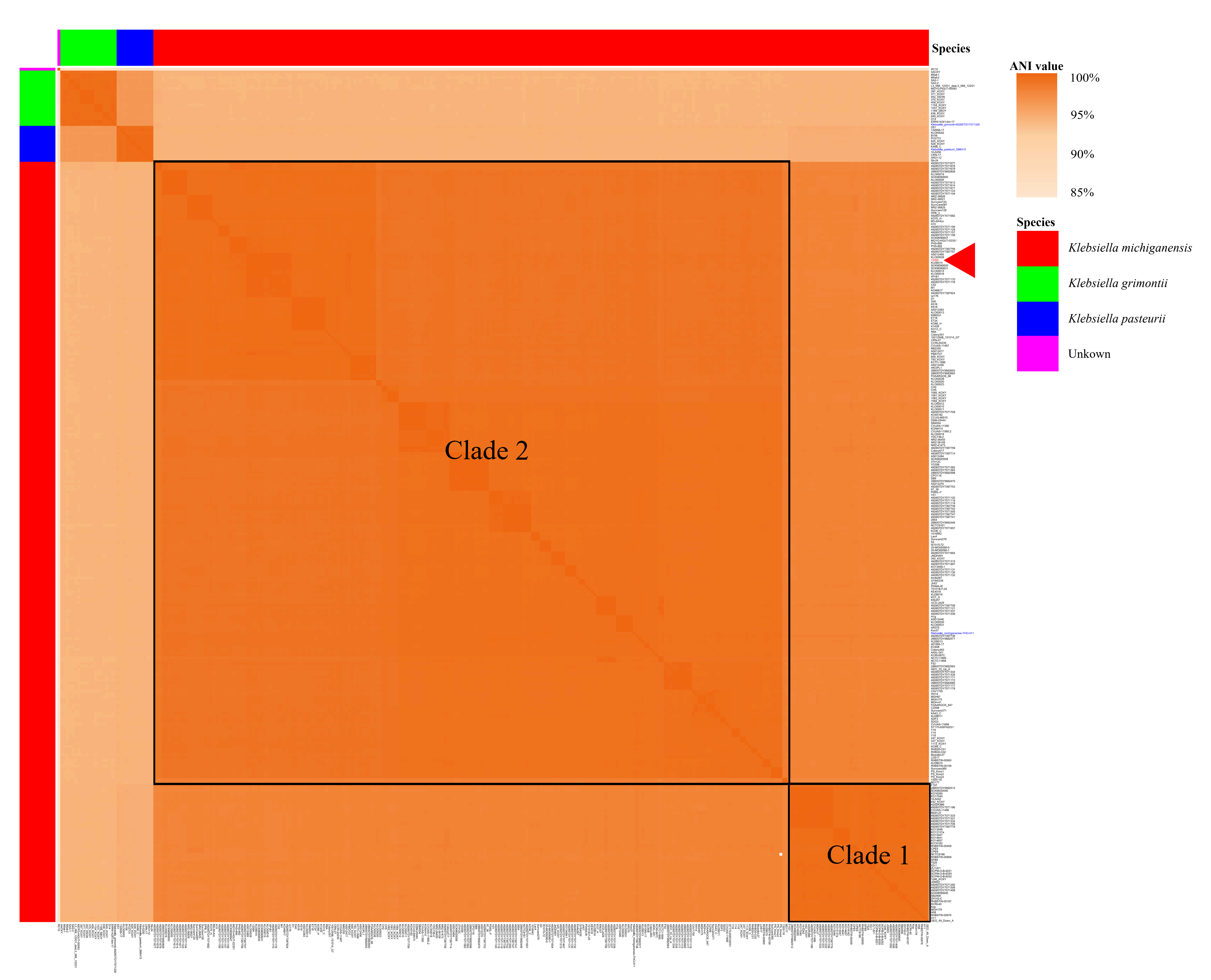

Supplement: Supplementary Figure 1 — Pairwise average nucleotide identity (ANI) cluster map of all the downloaded genomes with Klebsiella reference strains. Shown is the hierarchical clustering and heat map of pairwise ANI values among all strains. Their final species determination (K. michiganensis, K. grimontii, and K. pasteurii) are shown as colored bars adjacent to the heat map. The strain12084 is marked with a red triangle. [file DataSheet_1.zip › Supplementary materials/FIGURE S1.tif]

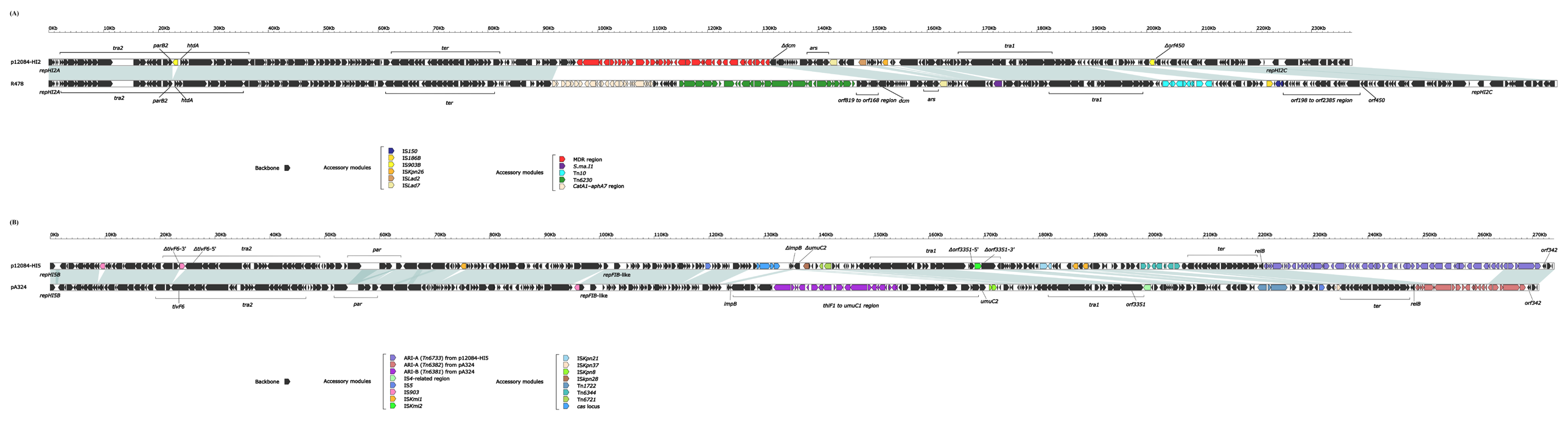

Supplement: Supplementary Figure 1 — Pairwise average nucleotide identity (ANI) cluster map of all the downloaded genomes with Klebsiella reference strains. Shown is the hierarchical clustering and heat map of pairwise ANI values among all strains. Their final species determination (K. michiganensis, K. grimontii, and K. pasteurii) are shown as colored bars adjacent to the heat map. The strain12084 is marked with a red triangle. [file DataSheet_1.zip › Supplementary materials/FIGURE S2.tif]

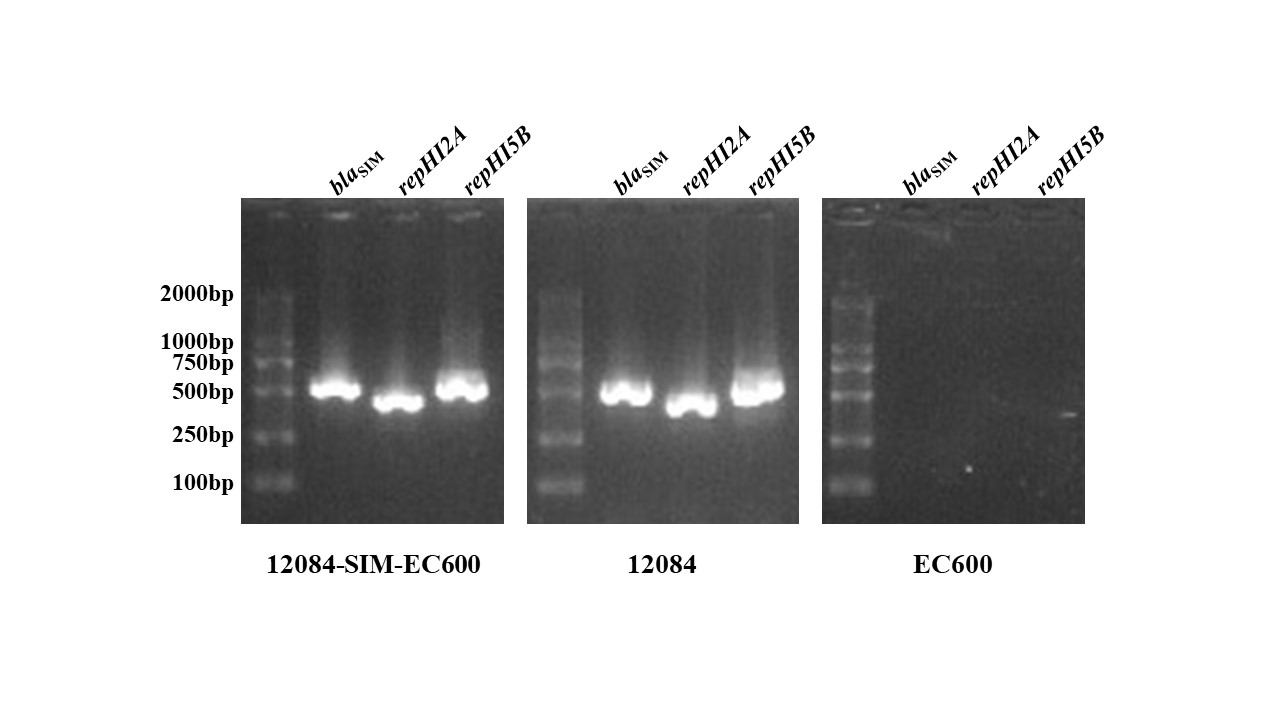

Supplement: Supplementary Figure 1 — Pairwise average nucleotide identity (ANI) cluster map of all the downloaded genomes with Klebsiella reference strains. Shown is the hierarchical clustering and heat map of pairwise ANI values among all strains. Their final species determination (K. michiganensis, K. grimontii, and K. pasteurii) are shown as colored bars adjacent to the heat map. The strain12084 is marked with a red triangle. [file DataSheet_1.zip › Supplementary materials/FIGURE S3.jpg]

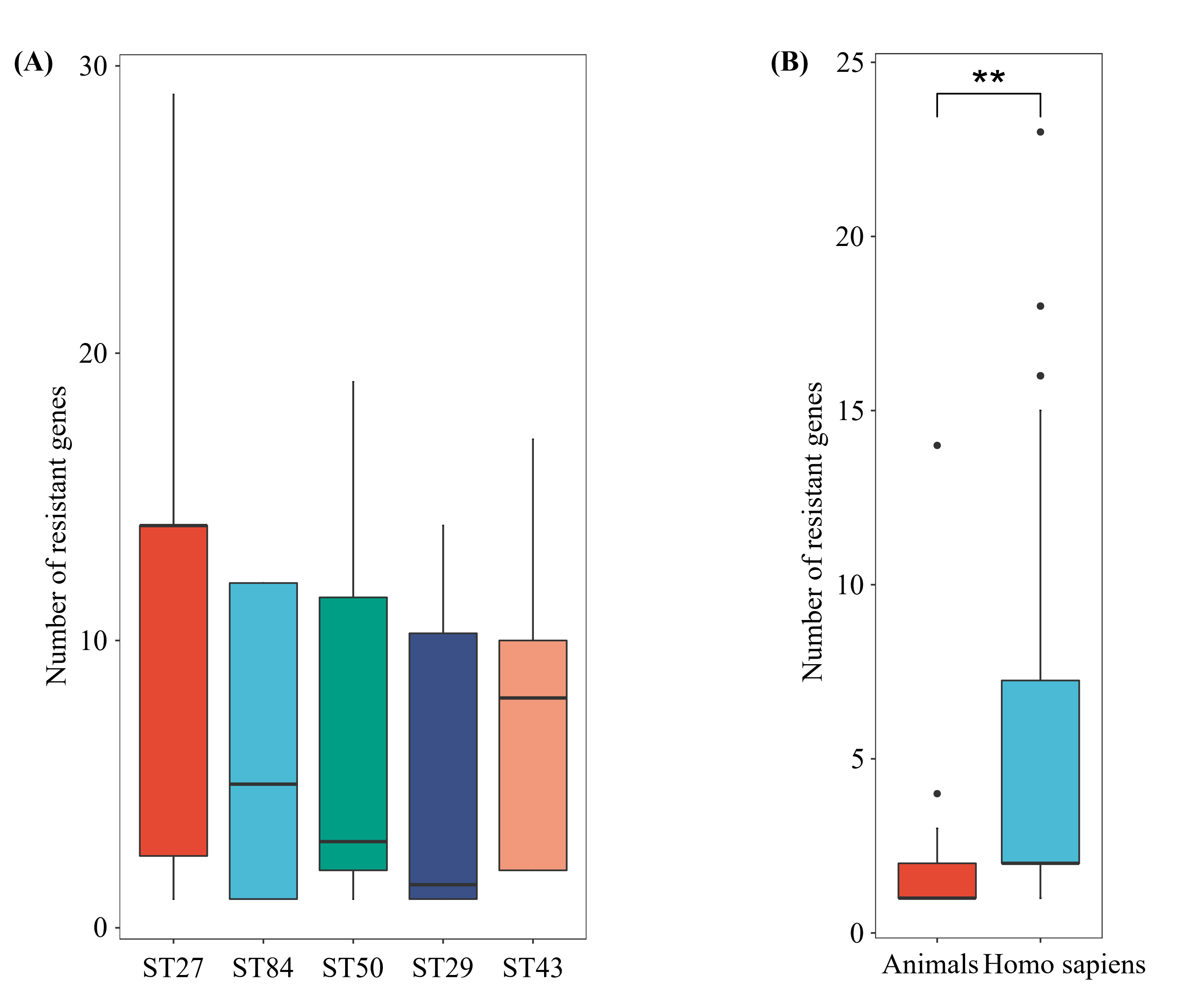

Supplement: Supplementary Figure 1 — Pairwise average nucleotide identity (ANI) cluster map of all the downloaded genomes with Klebsiella reference strains. Shown is the hierarchical clustering and heat map of pairwise ANI values among all strains. Their final species determination (K. michiganensis, K. grimontii, and K. pasteurii) are shown as colored bars adjacent to the heat map. The strain12084 is marked with a red triangle. [file DataSheet_1.zip › Supplementary materials/FIGURE S4.tif]
